# Aberrant age-related alterations in spontaneous cortical activity in participants with cerebral palsy

**DOI:** 10.3389/fneur.2023.1163964

**Published:** 2023-07-13

**Authors:** Hannah Bergwell, Michael P. Trevarrow, Elizabeth Heinrichs-Graham, Anna Reelfs, Lauren R. Ott, Samantha H. Penhale, Tony W. Wilson, Max J. Kurz

**Affiliations:** ^1^Institute for Human Neuroscience, Boys Town National Research Hospital, Omaha, NE, United States; ^2^Department of Pharmacology & Neuroscience, Creighton University, Omaha, NE, United States

**Keywords:** magnetoencephalography, resting state, sensorimotor, beta, neuroimaging

## Abstract

**Introduction:**

Cerebral Palsy (CP) is the most common neurodevelopmental motor disability, resulting in life-long sensory, perception and motor impairments. Moreover, these impairments appear to drastically worsen as the population with CP transitions from adolescents to adulthood, although the underlying neurophysiological mechanisms remain poorly understood.

**Methods:**

We began to address this knowledge gap by utilizing magnetoencephalographic (MEG) brain imaging to study how the amplitude of spontaneous cortical activity (i.e., resting state) is altered during this transition period in a cohort of 38 individuals with spastic diplegic CP (Age range = 9.80–47.50 years, 20 females) and 67 neurotypical controls (NT) (Age range = 9.08–49.40 years, Females = 27). MEG data from a five-minute eyes closed resting-state paradigm were source imaged, and the power within the delta (2–4 Hz), theta (5–7 Hz), alpha (8–12 Hz), beta (15–29 Hz), and gamma (30–59 Hz) frequency bands were computed.

**Results:**

For both groups, the delta and theta spontaneous power decreased in the bilateral temporoparietal and superior parietal regions with age, while alpha, beta, and gamma band spontaneous power increased in temporoparietal, frontoparietal and premotor regions with age. We also found a significant group x age interaction, such that participants with CP demonstrated significantly less age-related increases in the spontaneous beta activity in the bilateral sensorimotor cortices compared to NT controls.

**Discussion:**

Overall, these results demonstrate that the spontaneous neural activity in individuals with CP has an altered trajectory when transitioning from adolescents to adulthood. We suggest that these differences in spontaneous cortical activity may play a critical role in the aberrant motor actions seen in this patient group, and may provide a neurophysiological marker for assessing the effectiveness of current treatment strategies that are directed at improving the mobility and sensorimotor impairments seen in individuals with CP.

## 1. Introduction

Cerebral palsy (CP) is the most common neurodevelopmental motor disability, with recent population-based studies reporting prevalence estimates of 1–4 per 1,000 live births (1, 2). The majority of the cases with CP result from a pre−/peri- natal insult, with a small sub-class being of a genetic etiology. This group of disorders commonly results largely in movement, balance, and postural impairments, but is also characterized by impairments across sensory, perception, and cognitive domains (3). The scientific literature involving individuals with CP tends to focus on childhood and development, with little attention to the transition into adulthood (4–6). Even in individuals who are independently mobile in their youth and early adulthood, there is a marked decline in ambulation that often occurs in concert with premature aging (4). The clinical literature has reported that most adults with CP between the ages of 20 and 40 years will experience some form of premature aging, which is characterized by motor function decline, as well as increases in pain, fatigue, and decreased cognitive functioning (4, 7–10).

Resting-state neuroimaging studies provide an inclusive approach to examining brain activity that largely avoids the confounding factors of variable cognitive capacity, attentional span, and motor deficits that can impede task-based paradigms across patient populations (11). As such, neuroimaging studies utilizing resting-state paradigms offer a unique opportunity to gain insight into the neurophysiology underlying cognitive and sensorimotor function (12–14). Currently, fMRI is the most commonly used tool for investigating resting-state activity and the existing studies focusing on those with CP have largely examined functional connectivity [FC; (15–18)]. These investigations, all of which have evaluated adolescence and young adults with CP, have generally found that FC is altered across a myriad of networks compared with neurotypical (NT) controls (15, 16). Specifically, studies have shown that youth with spastic diplegic CP have altered FC in the sensorimotor network, frontoparietal network and salience network (17, 18). Other studies have found that youth with CP have increased FC in sensorimotor regions, and decreased FC between sensorimotor cortices and the cerebellum, visual and parietal cortices (15, 17–19). A recent fMRI study from our laboratory has also reported an association between decreased FC in the occipital and sensorimotor cortices and the extent of alterations in the gait biomechanics of individuals with CP (20). This indicates that alterations in FC exist widely in youth with CP and that resting-state paradigms may have direct relevance to assessing the motor and cognitive dysfunction that is observed within this population. As mentioned above, the aforementioned fMRI studies focused on youth and young adults with CP and there are no studies to date examining the trajectory of resting-state activity in aging adults with CP. Thus, the long-term impact of the initial brain insult on the underlying neurophysiology is poorly understood.

Of late, there has been a growing interest in utilizing other dimensions of resting-state neural activity, such as the spontaneous cortical activity measured via magnetoencephalography (MEG). MEG is an ideal tool for intellectually and developmentally disabled populations, as it is noninvasive, silent, and has high temporal resolution (~1 ms) and spatial precision (~3–5 mm; (21)). Spontaneous activity reflects the seemingly random neuronal discharges, fluctuations in dendritic currents, and other electrical field phenomena that occur across the cortex in the absence of exogenous and endogenous inputs and is often examined using a band-limited approach that focuses on the canonical frequency bands. Previous MEG and electroencephalography (EEG) studies have demonstrated that resting-state network dynamics change with advancing age in NT populations (22–29). A recent investigation that represents the largest lifespan MEG study to date (6–84 yrs., *N* = 434) has revealed that there are linear age-related changes in the relative spontaneous cortical activity across the conical frequency bands (28). Specifically, this investigation showed that the relative power decreases across the delta and theta frequency bands with age, while there are linear increases in the power of the alpha, beta and gamma frequency bands. Despite these insights, the field has yet to probe the potential age-related changes in the spontaneous activity of those with CP. Assessing the linear trajectory of the cortical spontaneous oscillations might shed new light on the functional declines seen across the adolescent to adulthood timeframe. Furthermore, it might indicate that there is a potential for an accelerated aging profile in this patient population.

The overall goal of this investigation was to evaluate the age-related changes seen in the spontaneous cortical activity of persons with CP. This goal was accomplished by evaluating the MEG eyes-closed resting-state recordings collected by Trevarrow and colleagues that initially identified that the spontaneous cortical activity for persons with CP is uncharacteristic when compared with NT controls (30). Since the clinical literature has reported that most adults with CP between the ages of 20 and 40 years will experience some form of premature aging, we hypothesized that the persons with CP would have aberrant age-related changes in the linear trajectory of their spontaneous cortical activity when compared to NT controls.

## 2. Methods

### 2.1. Participants

The Trevarrow et al. (30) dataset used in this investigation was comprised of 105 participants (30). Thirty-eight of the participants had spastic diplegic CP (GMFCS I-IV, Mean Age = 22.08 ± 10.46 yrs., Age Range = 9.80–47.50 yrs., Females = 20) and 67 were NT controls (Mean Age = 19.56 ± 10.25 yrs., Age Range = 9.08–49.40 yrs., Females = 27). Further details on the distribution of the participants are shown in the Supplementary information. The exclusion criteria for this dataset included any musculoskeletal surgeries in the past six months, botulinum toxin injections in the past year, anti-spastic medications and/or GABAergic medications. Botulinum toxin injections were considered as an exclusion criterion because prior studies have shown that these injections can influence the spontaneous cortical activity (31–33). In addition, none of the participants had a prior history of epilepsy or mood disorders. As indicated in Trevarrow et al. (30), informed consent was acquired, and the youth assented to participate in the experiment. Furthermore, the local Institutional Review Board reviewed and approved the study, and all protocols were in accordance with the declaration of Helsinki.

### 2.2. MEG data acquisition, source imaging and frequency power maps

Complete details of the MEG acquisition, pre-processing and source imaging are found in Trevarrow et al. (30). Briefly, a 306-sensor Elekta/MEGIN MEG system (Helsinki, Finland) was used to sampled continuously at 1 kHz as the participants completed a resting state paradigm with their eyes closed for 5 minutes. MEG data processing was completed in Brainstorm (34) and largely followed the analysis procedure outlined in (35, 36). A high pass filter of 0.3 Hz and notch filters at 60 Hz and at its harmonics were applied. Cardiac and eye movement artifacts were identified in the raw MEG data and removed using an adaptive signal-space projection (SSP) approach, which was subsequently accounted for during source reconstruction (37). Data were then divided into four-second epochs for detection and rejection of bad segments of data based on amplitude and gradient distributions per participant. There were no statistical differences in the number of epochs accepted for the respective groups (CP = 63.63 ± 5.33 epochs; NT = 63.88 ± 5.63 epochs; *p* = 0.822). On average, 83% of the epochs were included for the respective groups.

As in Trevarrow et al. (30), minimum norm estimates were computed and normalized by a dynamic statistical parametric mapping (dSPM) algorithm for source imaging. An empty room recording to compute a noise covariance matrix for source imaging was utilized to account for environmental noise (38). The forward model was computed using an overlapping spheres head model (39). Finally, the imaging kernel of depth-weighted dSPM constrained to the individual cortical surface (40) was computed. Using the resulting source estimates, the power of cortical activity in the delta (2–4 Hz), theta (5–7 Hz), alpha (8–12 Hz), beta (15–29 Hz), and gamma (30–59 Hz) frequency bands were computed. Welch’s method with 1 second sliding Hamming windows overlapping at 50% was used to estimate the power spectrum densities (PSD) on each four-second epoch for each MEG recording. To create relative maps, we standardized the PSD values at each frequency bin to the total power across the frequency spectrum. For each participant, we then averaged PSD maps across epochs to obtain one set of PSD maps per participant. Finally, we projected these maps onto the MNI ICBM152 brain template when scaling was used during coregistration (41) and applied a 3 mm full width half max (FWHM) smoothing kernel. The resulting normalized source maps per frequency band were used for further statistical analysis.

### 2.3. Statistical analyses

We analyzed the whole-brain PSD maps in SPM12 to examine for spatially specific effects of age and group (CP vs. NT). For each frequency band, we ran an ANCOVA with group as a categorical predictor and age as a continuous predictor and modeled the respective interaction term. To correct for multiple comparisons, we applied threshold free cluster enhancement [TFCE; (42)] with a weighting factor of *E* = 0.6 and a cluster level family wise error (FWE) of 0.05 to the resulting statistical maps. Finally, TFCE maps were thresholded by utilizing the clusters that survived correction. Data from each of the clusters were averaged and used to display the corresponding effects. Note that the TFCE log transformed values are reported in the results.

## 3. Results

### 3.1. Main effect of group

The group main effect was similar to what is presented in Trevarrow et al. (30), where the individuals with CP had significantly stronger delta power with peaks in the left (CP = 30.8 ± 1.4%, NT = 26.6 ± 1.0%, TFCE = 5.63, *p_FWE_* = 0.013) and right (CP = 31.4 ± 1.3%, NT = 27.0 ± 1.0%, TFCE = 5.64, *p_FWE_* = 0.013) occipital areas compared to NT controls. Within the theta band, participants with CP had significantly stronger activity in the left occipital region (CP = 21.7 ± 1.0%, NT = 19.1 ± 0.6%, TFCE = 4.44, *p_FWE_* = 0.037). In contrast, controls had significantly stronger alpha activity, with peaks in the left (CP = 31.4 ± 2.1%, NT = 38.5 ± 1.7%, TFCE = 5.57, *p_FWE_* = 0.014) and right occipital (CP = 32.0 ± 0.6%, NT = 39.2 ± 1.7%, TFCE =5.50, *p_FWE_* = 0.016) areas, as well as the right prefrontal region (CP = 15.9 ± 0.6%, NT = 18.3 ± 0.6%, TFCE = 4.97, *p_FWE_* = 0.029) compared to participants with CP. In the beta band, participants with CP had increased power compared to controls in the left secondary somatosensory cortical area (CP = 9.2 ± 0.3%, NT = 7.9 ± 0.2%, TFCE = 5.46, *p_FWE_* = 0.016). Finally, in the gamma band, participants with CP had significantly stronger spontaneous activity relative to NT controls in both the left (CP = 4.8 ± 0.3%, NT = 3.5 ± 0.1%, TFCE = 7.02, *p_FWE_* < 0.001) and right SII regions (CP = 4.5 ± 0.2%, NT = 3.4 ± 0.1%, TFCE = 6.23, *p_FWE_* < 0.001).

### 3.2. Main effect of Age

**Figure 1:**
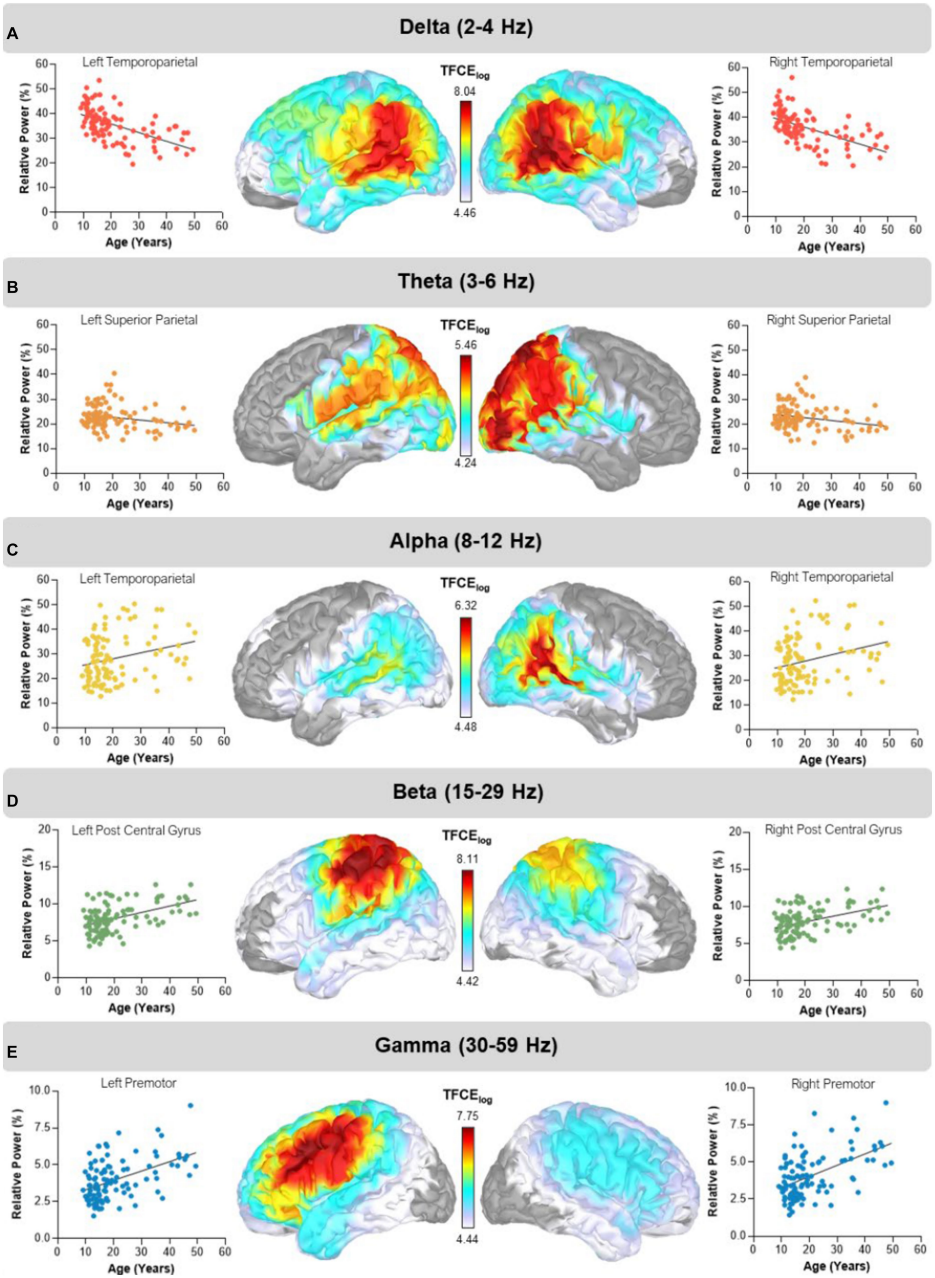
Main effects of age. Statistical maps thresholded with threshold-free cluster enhancement (TFCE) depict regions showing aging effects in the delta, theta, alpha, beta, and gamma bands. The corresponding scatter plots display relative power in percent units on the y-axis and age on the x-axis. The dots and trendline in each plot represent extracted values from each cluster peak per participant. **(A)** Delta (2–4 Hz) power decreased with advancing age in bilateral temporoparietal regions. **(B)** Theta (3–6 Hz) power decreased with advancing age in bilateral superior parietal regions. **(C)** Alpha (8–12 Hz) power increased with advancing age in bilateral temporoparietal regions. **(D)** Beta (15–29 Hz) power increased with advancing age in the left postcentral gyrus and right superior parietal regions. **(E)** Gamma (30–59 Hz) band power increased with advancing age with bilateral cluster peaks in premotor regions. The color bar next to each map shows the log transformed TFCE values.

We found widespread aging effects in the delta range across both hemispheres, with peaks in the left (TFCE = 8.02, *p_FWE_* < 0.001; Figure 1A) and right (TFCE = 8.40, *p_FWE_* < 0.001; Figure 1A) temporoparietal regions, indicating that power decreased with advancing age. Similarly, in the theta band, spontaneous power decreased with advancing age, with peaks in the left (TFCE = 5.39, *p_FWE_* = 0.017) and right (TFCE = 5.46, *p_FWE_* = 0.014) superior parietal regions (Figure 1B). Conversely, spontaneous power in the alpha band significantly increased as a function of age with peaks in the left (TFCE = 6.11, *p_FWE_* = 0.005) and right (TFCE = 6.32, *p_FWE_* = 0.002) temporoparietal regions (Figure 1C). Likewise, beta power also increased with advancing age with peaks in the left postcentral gyrus (TFCE = 8.11, *p_FWE_* < 0.001) and right superior parietal region (TFCE = 7.96, *p_FWE_* < 0.001; Figure 1D). Finally, spontaneous gamma power increased with advancing age, with peaks in the left (TFCE = 7.75*p_FWE_* < 0.001) and right (TFCE = 7.27, *p_FWE_* < 0.001) premotor regions (Figure 1E).

### 3.3. Group x age interaction

**Figure 2:**
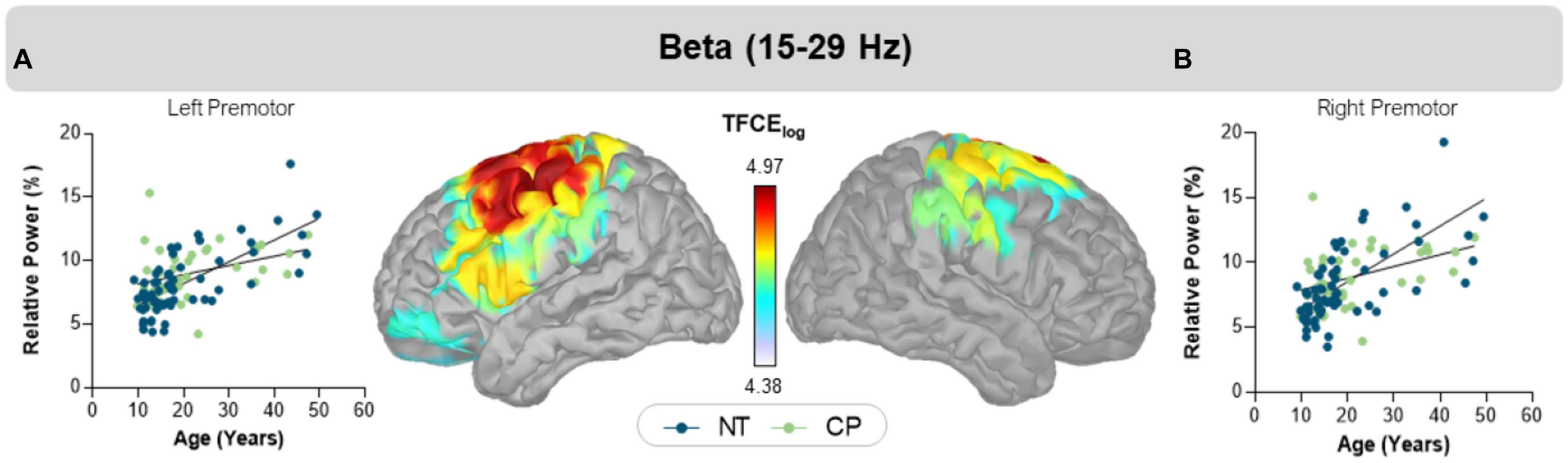
Interaction effects of group and age. Scatter plots display relative beta power in percent units on the y-axis and age on the x-axis in bilateral left **(A)** and right **(B)** motor cortices. The blue circles and trendline represent NT controls, while the green circles and trendline represent participants with CP. In both regions, relative beta power increased in both groups, but with distinct slopes. The color bar between the two maps shows the scale of log transformed TFCE values. Extracted cluster peak average values are plotted for each participant in the graph.

We observed a group-by-age interaction in the beta band, with peaks in the left (CP = 9.0 ± 0.3%, NT = 8.0 ± 0.3%, TFCE = 4.97, *p_FWE_* = 0.032) and right (CP = 9.0 ± 0.3%, NT = 8.0 ± 4.0%, TFCE = 4.79, *p_FWE_* = 0.039) motor areas (i.e., precentral gyrus, supplemental motor area, and premotor cortices; Figure 2). In each of these regions, the NT controls (*b* = 0.171; 95% CI = 0.129–0.213) showed a steeper increase in beta power with age than the participants with CP (*b* = 0.072; 95% CI = 0.008–0.137). No other age by group interactions were detected using our statistical approach.

## 4. Discussion

Our results demonstrated that the strength of spontaneous activity changes with age for both those with CP and NT controls, and that the location of such changes varies by spectral band. Furthermore, the central hypothesis of this study was also supported, as we uncovered an altered age-related trajectory of beta power within the sensorimotor cortices in the participants with CP. Although the perinatal injury that persons with CP experience is defined as not being progressive (3), this finding suggests that the early perinatal brain injury may have an impact on spontaneous activity across the life span. We speculate that the psychosocial stress and/or adversity in the early years of life of those with CP might result in a heightened inflammatory response that can have long-term influence on the spontaneous cortical activity (43, 44). In the following sections we will further discuss the implications of the current findings.

Our findings revealed robust age-related findings across all canonical frequency bands for both groups. Specifically, we found that delta and theta spontaneous power decreased in bilateral temporoparietal and superior parietal regions, respectively. Conversely, we found that alpha, beta, and gamma band spontaneous power increased in temporoparietal, frontoparietal and premotor regions, respectively. This aligns with previous work which has shown a decrease in power in slow wave frequencies such as delta and theta, and an increase in power in faster wave frequencies such as alpha, beta, and gamma with age (22, 26, 28). Recent work has further shown that this shift from low to high frequencies with age in a cohort of NT youths across development, finding widespread decreases in delta power and more focal increases in alpha, beta, and gamma power across development (36).

Our results also show that persons with CP have less prominent changes in the sensorimotor beta power with age than what is seen in the NT population. Our prior experimental results have overwhelmingly shown that task-based beta oscillations are aberrant when participants with CP plan and execute a motor action (45–47). Furthermore, Heinrichs-Graham and colleagues have revealed that the strength of beta sensorimotor cortical oscillations during movement in older adults is tightly linked with the spontaneous beta power in the same cortical tissue (23, 24). Taken together, it is possible that similar age dependent changes in the spontaneous cortical oscillations may play a critical role in the aberrant task-based beta cortical oscillations seen in individuals with CP. Further exploration of the potential link between the strength of task-based and spontaneous sensorimotor cortical oscillations are warranted, as such studies have the potential to further illuminate the neurophysiological mechanisms underlying the altered motor actions seen in this patient population.

Mechanistically, beta cortical oscillations are thought to be modulated by γ-aminobutyric acid (GABA) activity. Prior pharmaco-MEG studies have shown that spontaneous beta amplitude in the sensorimotor cortices increases with the administration of a GABA_A_ receptor agonist (48) or when a GABA transporter is blocked (49). GABAergic activity intrinsically plays a role in beta band power, therefore the increased strength of spontaneous beta activity may be due to heightened activity of GABAergic inhibitory interneurons (50). Previous PET investigations showed that individuals with CP have increased GABA_A_ receptor binding potential within the motor cortices (51). These findings overlap with the age-related findings in beta power discussed above, suggesting that increased GABA activity in NT older adults as well as individuals with CP, could be driving the age-related effects seen in motor cortices in the beta frequency band.

There has been a growing interest in the use of MEG to assess the spontaneous cortical oscillations, with intentions of identifying clinically relevant biomarkers (28, 30, 52, 53). Prior experimental work has shown that MEG assessments of the spontaneous relative power of the respective frequency bands are reliable within the week and across several years (54, 55). Together these studies have reported that the MEG assessments of the spontaneous relative power are highly reliable for the theta, alpha and beta frequency bands. This infers that the groupwise differences seen across the spontaneous theta, alpha and beta frequency bands, and age-related changes seen in the beta oscillations for the persons with CP are robust. We also anticipate that the results presented here will be repeatable in subsequent studies for two reasons. For one, the alpha and beta oscillations are two of the dominant cortical rhythms that are tightly connected with sensorimotor and cognitive control. Secondarily, our methodology used an established, reproducible and open-source pipeline for assessing the spontaneous cortical activity (56, 57). As such, we anticipate that the next generation of studies will find very similar results as what is reported here.

### 4.1. Limitations

Although this investigation provides unique insights on the neurophysiology of persons with CP, it should be recognized that the noted differences are related to the spontaneous cortical activity and not the cortical activity that would be associated with processing stimuli or the production of a motor action. That being said, prior investigations have shown that the spontaneous activity impacts the degree of the cortical activity that would induced by stimuli (23, 24). Furthermore, it should be noted that although some participants may not be able to complete task-based paradigms due to cognitive or motor constraints (i.e., spasticity, contractions, etc.), almost all participants can sit quietly in the MEG to assess the spontaneous cortical activity. Hence, the spontaneous methodology employed here does have advantages. Other limitations include the use of a cross-sectional design to evaluate the age-related changes in the spontaneous cortical activity as persons with CP transition from adolescents to adults. Although this approach is convenient, it does not allow for the evaluation of the individual longitudinal changes seen across this important time window. Furthermore, the approach employed in this investigation only focused on the age-related differences seen in the relative power of the respective cortical oscillations and did not examine the connectivity amongst the respective areas. We propose that such analysis will provide a more comprehensive understanding of how brain networks are impacted as adolescents with CP transition into adulthood. This investigation was MEG centric and did not include a complementary sensory and motor assessments, which limits our ability to more fully establish how aberrations in the respective frequency bands are related to the clinical declines seen in persons with CP as they transition into adulthood. Lastly, since the cohort included in this investigation predominantly spanned the adolescent to adult age range it still remains unknown if the noted altered spontaneous activity maintains the same trajectory as persons enter late adulthood.

## 5. Conclusion

In summary, our study found age dependent changes in spontaneous cortical activity in participants with CP and NT controls during the transition from adolescence to adulthood. Our age level analyses replicated previous literature with a general weakening of slower frequencies and a strengthening of higher frequencies. Most significantly, we found an altered age-related trajectory in the beta band in the bilateral motor cortices, such that beta band power increased more gradually with age in the individuals with CP compared to their NT peers. We predict that greater aberrations in the spontaneous motor beta oscillations are partially linked with the accelerated motor declines seen in adults with CP. Overall, these results provide new insight on how the perinatal brain injuries seen in persons with CP impacts their cortical physiology. Moreover, we speculate that the altered trajectory of spontaneous cortical activity may contribute to the progressive decline in motor function with age that is often reported clinically.

## Data availability statement

The raw data supporting the conclusions of this article will be made available by the authors, without undue reservation.

## Ethics statement

The studies involving human participants were reviewed and approved by Boys Town Research Hospital’s Institution Review Board. Written informed consent to participate in this study was provided by the participants’ legal guardian/next of kin.

## Author contributions

HB contributed to data processing, analysis, interpretation of results and construction of original manuscript draft. MT contributed to the data processing, analysis, interpretation of results and manuscript revisions. EH-G contributed to the study design, interpretation of the results and manuscript revisions. AR, LO, and SP contributed to the data processing and manuscript revisions. TW and MK contributed to the study design, formulation of the hypotheses, guiding the data processing and statistical analyses, interpretation of the results and revision of the manuscript. All authors contributed to the article and approved the submitted version.

## Funding

This work was partially supported by funding from the National Institutes of Health (R01HD101833, R01HD108205, P20GM144641).

## Conflict of interest

The authors declare that the research was conducted in the absence of any commercial or financial relationships that could be construed as a potential conflict of interest.

## Publisher’s note

All claims expressed in this article are solely those of the authors and do not necessarily represent those of their affiliated organizations, or those of the publisher, the editors and the reviewers. Any product that may be evaluated in this article, or claim that may be made by its manufacturer, is not guaranteed or endorsed by the publisher.
