# Supplementary material for: Aberrant age-related alterations in spontaneous cortical activity in participants with cerebral palsy
Source: Front Neurol. 2023 Jul 13;14:1163964. doi: 10.3389/fneur.2023.1163964 (PMC10374009; doi:10.3389/fneur.2023.1163964)
Supplement: Supplementary file 1 [file Data_Sheet_1.PDF]

Table 1. Participant Demographics. GMFCS= Gross Motor Classification Score, SD = Standard deviation

Group	Number Participants	Age Mean(SD)	Age Range	Number Females	GMFCS Range
Cerebral Palsy	38	22.08± 10.46 yrs.	9.90-47.50 yrs.	20	I-IV
Neurotypical Controls	67	19.58±10.25 yrs.	9.08-49.40	27	

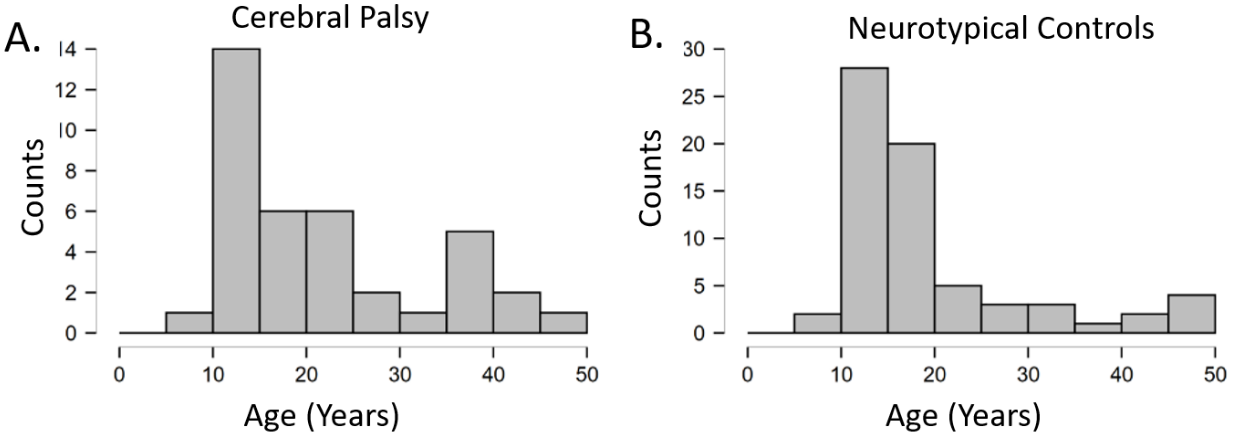

Figure 1. Age distributions for the participants with cerebral palsy and the neurotypical controls.

Table 2. Summary of the statistical results and location of the peak cortical activity for the respective frequency bands. CP = Cerebral Palsy, NT = Neurotypical. The Threshold Free Cluster

Enhancement (TFCE) are presented as log transformed values, and a cluster level family wise error (FWE) of 0.05 was applied to the statistical maps to identify significant differences. The location represents the MNI coordinates of the peak voxel in the cluster.

Main Effect of Group

	CP	NT	TFCE	p_{FWE}	Peak Location
Delta					
Left Occipital	30.8±1.4%	26.6+1.0%	5.63	0.013	-7,-101,-2
Right Occipital	31.4+1.3%	27.0+1.0%	5.64	0.013	5,-95,-4
Theta					
Left Occipital	21.7±1.0%	19.1±0.6%	4.44	0.037	-5,-103,1
Alpha					
Left Occipital	31.4±2.1%	38.5±1.7%	5.57	0.014	-9,-101,4
Right Occipital	32.0±0.6%	39.2±1.7%	5.50	0.016	17,-103,-6
Right Prefrontal	15.9±0.6%	18.3±0.6%	4.97	0.029	-27,65,-12
Beta					
Left SII	9.2±0.3%	7.9±0.2%	5.46	0.016	-63,-19,18
Gamma					
Left SII	4.8±0.3%	3.5±0.1%	7.02	<0.001	-67,-24,30
Right SII	4.5±0.2%	3.4±0.1%	6.23	<0.001	66,-20,39

Group X Age Interaction

	CP	NT	TFCE	p_{FWE}	
Beta					
Left Motor Area	9.0±0.3%	8.0±0.3%	4.97	0.032	-5,7,63
Right Motor Area	9.0±0.3%	8.0±4.0%	4.79	0.039	42,-12,67

Main Effect of Age

	TFCE	p_{FWE}	Peak Location
Delta			
Left Temporoparietal	8.02	<0.001	-62,-51,20
Right Temporoparietal	8.40	<0.001	48,-47,15
Theta			
Left Superior Parietal	5.39	0.017	-3,-74,52
Right Superior Parietal	5.46	0.014	4,-74,50
Alpha			
Left Temporoparietal	6.11	0.005	-53,-50,19
Right Temporoparietal	6.32	0.002	52,-41,19
Beta			
Left Postcentral Gyrus	8.11	<0.001	-36,-38,48
Right Superior Parietal	7.96	<0.001	19,-51,60
Gamma			
Left Premotor	7.75	<0.001	-37,5,41
Right Premotor	7.27	<0.001	45,-23,38
